# Evaluation of diagnostic agreement between STANDARD E TB-Feron ELISA and TB-Feron FIA for tuberculosis infection in prison settings in Paraguay

**DOI:** 10.3389/fpubh.2026.1697667

**Published:** 2026-02-05

**Authors:** Sarita Aguirre, Julieta Mendez, Patricia Ríos, Analía Ortiz, Cynthia Céspedes, Ruth Lezcano, Angelica Medina, Renate Henning, Silvina Monteverde, Nestor Moreno, Gladys Estigarribia, Guillermo Sequera

**Affiliations:** 1 Programa Nacional de Control de la Tuberculosis, Ministerio de Salud Pública y Bienestar Social, Asunción, Paraguay; 2 Instituto Regional de Investigación en Salud, Universidad Nacional de Caaguazú, Coronel Oviedo, Paraguay; 3 Research Department, Universidad Central del Paraguay, Pedro Juan Caballero, Paraguay; 4 Diaz Gill Laboratorio, Asunción, Paraguay; 5 Catedra de Salud Pública, Facultad de Ciencias Médicas, Universidad Nacional de Asunción, Asunción, Paraguay

**Keywords:** agreement tests, interferon-gamma, Paraguay, prisons, tests, tuberculosis, tuberculosis infection

## Abstract

**Introduction:**

Accurate diagnosis of tuberculosis (TB) infection is essential for TB control, particularly in high-risk populations. Interferon-gamma release assays (IGRAs) are widely used for TB infection screening. This study assesses the diagnostic agreement between the STANDARD™ F TB-Feron fluorescence immunoassay (FIA) and the WHO-approved STANDARD E TB-Feron ELISA in penitentiary settings in Paraguay.

**Methods:**

A cross-sectional study was conducted among 737 participants (642 incarcerated individuals and 95 staff members). Both assays were performed on the same blood samples from each participant. Agreement was assessed using the Kappa coefficient, with stratified analyses across predefined risk groups: G1, no known TB exposure (*n* = 71); G2, TB exposure (*n* = 525); G3, active TB (*n* = 67); and G4, previously treated and cured TB (*n* = 74).

**Results:**

Overall positivity was 57.7% with FIA and 56.9% with ELISA. Positivity rates for FIA/ELISA were: G1 (22.9%/22.9%), G2 (57.7%/58.4%), G3 (79.1%/79.1%), and G4 (71.6%/71.6%). Overall agreement was excellent (κ = 0.851; 95% CI: 0.812–0.889; *p* < 0.0001). Stratified analyses showed moderate agreement in G1 (κ = 0.595) and excellent agreement in the remaining groups.

**Conclusion:**

The STANDARD F TB-Feron FIA demonstrated excellent concordance with ELISA, supporting its use as a reliable alternative for TB infection screening. In addition, the FIA offers key operational advantages, including reduced hands-on time (approximately 15 min compared with several hours for ELISA), the ability to process single samples without batch runs, and the use of portable equipment requiring less laboratory infrastructure, making it particularly suitable for decentralized and resource-limited settings.

## Introduction

Tuberculosis (TB), caused by *Mycobacterium tuberculosis*, remains one of the leading causes of death from a single infectious agent worldwide. Despite advances in diagnosis and treatment, the World Health Organization (WHO) estimates that approximately one-quarter of the global population is living with TB infection, representing a vast reservoir for future cases of active TB (1, 2). The control and eventual elimination of TB critically depend on the capacity of health systems to effectively identify and treat individuals with TB infection—particularly among high-risk populations—to prevent progression to active disease (3, 4).

For decades, TB infection diagnosis relied on the tuberculin skin test (TST), which is known to have several limitations, including cross-reactivity with the BCG vaccine and non-tuberculous mycobacteria, and the requirement for a second visit to interpret the result (5, 6). Interferon-gamma release assays (IGRAs) emerged as a more specific alternative, detecting the cellular immune response to *M. tuberculosis*-specific antigens such as ESAT-6 and CFP-10. ELISA-based IGRA platforms, including QuantiFERON-TB Gold Plus and STANDARD E TB-Feron ELISA, are among the most widely used and have been endorsed by WHO for TB infection detection in high-risk populations (7, 8). However, these technologies require laboratory infrastructure, trained personnel and batch processing which may hinder their use in low-resource settings where TB is often more prevalent.

In recent years, several new diagnostic tests for tuberculosis (TB) infection have been rapidly developed. The STANDARD E TB-Feron ELISA (SD Biosensor) has been recently recommended by the World Health Organization (WHO) as a valid tool for TB infection detection. It is important to note, however, that no definitive gold standard exists for TB infection diagnosis, as the infection state cannot be directly observed; therefore, ELISA-based interferon-gamma release assays (IGRAs) are commonly used as reference comparators in validation studies. The STANDARD F TB-Feron FIA (SD Biosensor), a fluorescence-based immunoassay, has been proposed as an alternative to ELISA, offering several operational advantages during the detection phase. While both assays share identical pre-analytical requirements—including blood collection in specialized tubes, incubation at 37 °C for 16–24 h, and plasma separation by centrifugation—key differences arise in analytical workflow. The FIA enables single-sample processing without the need for batch runs, substantially reduces hands-on laboratory time (approximately 15 min from plasma loading to result compared with 3–4 h for ELISA), and relies on compact, portable equipment rather than conventional microplate readers and extensive laboratory infrastructure. These features may facilitate decentralized TB infection testing and support timely contact tracing in resource-limited settings. Nevertheless, it is important to emphasize that FIA is not a true point-of-care test, as it still requires incubation, centrifugation, and trained personnel; thus, its use is better described as decentralized testing in peripheral health facilities rather than bedside or field-based diagnostics (9, 10).

Despite the operational advantages of FIA-based technology, its diagnostic performance must be validated against established tests. Recent studies have reported high concordance between TB-Feron FIA and ELISA in settings such as Eastern Europe and Asia (11, 12). However, evidence is lacking on the performance of these tests in South American populations, particularly among persons deprived of liberty (PDL). Prisons represent critical settings for TB control due to overcrowding, poor ventilation, and delayed diagnosis, resulting in TB prevalence rates that far exceed those in the general population (13). Paraguay is considered a country with a moderate TB burden, with incidence rates ranging between 40 and 50 cases per 100,000 populations over the past 5 years (1). Although persons deprived of liberty (PDL) represent only 0.22% of the national population (14), they account for 20% of the country's annual TB cases, with incidence rates exceeding 3,500 per 100,000—over 70 times higher than that of the general population (15). The incidence ratio between male and female prisons is greater than 5:1, compared to over 2:1 in the general population (15). The validation of rapid and reliable diagnostic tools in such high-transmission environments is essential for improving TB infection screening and preventive therapy programs.

Therefore, the objective of this study was to evaluate the diagnostic agreement between the STANDARD F TB-Feron FIA and the reference STANDARD E TB-Feron ELISA for the detection of TB infection in a high-risk population, composed of incarcerated individuals and prisons staff in Paraguay.

## Methods

We conducted a cross-sectional diagnostic test evaluation study between June and August 2023 in two penitentiary centers (PC) in Paraguay. The cross-sectional design was selected to enable simultaneous evaluation of both assays under real-world field conditions, without the need for longitudinal follow-up.

The study was conducted in male and female PCs in Coronel Oviedo and in the PC of Ciudad del Este. Participants were recruited through consecutive sampling, during the study period. All eligible PDL and prison staff who met the inclusion criteria were approached and invited to participate. Inclusion critera were: adults aged ≥18 years who voluntarily agreed to participate and provided written informed consent. Individuals were stratified into four mutually exclusive risk groups based on their TB exposure history and clinical status: **Group 1** (Low Risk): Individuals with no documented history of close contact with active TB cases. While we acknowledge that TB transmission may occur in prison settings, participants in this group had no identified exposure through contact investigation or clinical records and were not under investigation for TB symptoms at the time of enrollment. **Group 2** (High Risk): identified contacts of active TB cases, with high risk of progression to active TB. **Group 3** (Confirmed TB): individuals with bacteriologically confirmed active TB at the time of recruitment. **Group 4** (Recent TB History/Previous TB): individuals with a previous episode of bacteriologically confirmed TB treated within the past year. Additional data were collected on relevant risk factors, including diabetes, HIV status, tobacco use, and drug consumption, which were obtained through participant self-report.

Both assays detect interferon-gamma (IFN-γ) release in response to M. tuberculosis-specific antigens (ESAT-6, CFP-10, and TB7.7) and share the same blood collection tubes and incubation protocol, but differ in the detection method and workflow. Each participant provided 8 mL of blood, distributed into four specialized collection tubes: one Nil control tube (negative control), one Mitogen tube (positive control), and two TB Antigen tubes containing ESAT-6, CFP-10, and TB7.7 peptides. Following collection, all tubes were incubated at 37 °C for 16 to 24 h to stimulate IFN-γ production by T-cells in response to the antigens. After incubation, tubes were centrifuged to separate plasma from cellular components. The same plasma samples were then used for both assays.

STANDARD E TB-Feron ELISA: This assay uses enzyme-linked immunosorbent assay (ELISA) technology. Plasma samples are processed in batch format using microplate wells coated with anti-IFN-γ antibodies. The assay involves multiple manual pipetting steps, incubation periods, and washing cycles. IFN-γ concentration is quantified through colorimetric detection using a standard ELISA plate reader. The entire process, from plasma loading to final result interpretation, requires approximately 3–4 h of hands-on laboratory time and is typically performed in batches.

STANDARD F TB-Feron FIA: This assay employs fluorescence immunoassay (FIA) technology using the STANDARD F2400 automated reader (SD Biosensor, Gyeonggi-do, Republic of Korea), a portable and automated FIA platform. Plasma samples are loaded into single-use cartridges and inserted into the device, which automatically performs IFN-γ quantification through fluorescence detection and delivers results in approximately 15 min per sample. The FIA platform supports both single-sample testing without the need for batch processing and high-throughput workflows, as the F2400 reader can process up to 24 samples simultaneously. Although ELISA assays can technically be performed on individual samples, they are generally run in batches for economic and operational efficiency. This capability of the FIA reduces waiting times for result availability when multiple patients are tested, while requiring minimal hands-on time and utilizing a compact, portable reader.

Both assays were performed by trained laboratory personnel of Diaz Gill Laboratory, a certified laboratory in Paraguay following manufacturer's standardized protocols and under the supervision of the National TB Program reference laboratory (16, 17).

### Statistical analysis

The primary analysis focused on evaluating the agreement and concordance between the qualitative results of the TB-Feron ELISA and TB-Feron FIA assays. Given the absence of a definitive gold standard for TB infection diagnosis, we assessed concordance rather than true diagnostic accuracy. The STANDARD E TB-Feron ELISA was used as the reference comparator, as it is endorsed by WHO for TB infection detection (8).

In addition to the description of demographic variables and the proportion of positive cases for each test, Cohen's Kappa coefficient (κ) was used to assess concordance (18). The strength of agreement was interpreted as follows: < 0.20 (poor), 0.21–0.40 (fair), 0.41–0.60 (moderate), 0.61–0.80 (substantial), and 0.81–1.00 (excellent or almost perfect) (19). Ninety-five percent confidence intervals (95% CI) were calculated for the Kappa estimates. Concordance analysis was conducted for the overall study population and stratified by predefined risk groups, as well as by individual risk factors (sex, diabetes, HIV status, tobacco use, and drug use). Quantitative response values to TB antigen were also analyzed for both assays. The Pearson correlation coefficient was used to assess the association between the quantitative IFN-γ values obtained by the two assays. In addition, receiver operating characteristic (ROC) curves were generated for both tests as a comparative analytical approach to evaluate their ability to discriminate between predefined clinical risk groups using IFN-γ response levels. Given the absence of a definitive gold standard for TB infection, ROC analyses were not intended to estimate diagnostic accuracy *per se*, but to provide complementary information on the relative distribution and discriminatory performance of quantitative signals across relevant clinical categories. For these analyses, values exceeding 10 IU/mL were capped at 10 IU/mL. Concentrations above this threshold accounted for less than 10% of positive measurements and represented markedly elevated IFN-γ responses that do not add further interpretative value, as they already correspond to clearly positive results. Capping was applied to reduce the influence of extreme values on correlation analyses, while preserving the qualitative interpretation of test results, which is based on the combined assessment of all assay tubes. Participants with indeterminate results in either assay were excluded from concordance calculations, as these results cannot be classified as positive or negative and therefore do not contribute to agreement assessment. The data were analyzed using STATA 18.0 statistical software.

### Ethical considerations

All participants provided written informed consent for study participation and blood collection. Results from the WHO approved test were interpreted according to national TB guidelines, and treatment was initiated as recommended by the National TB Program. Data were anonymized for the purpose of test concordance analysis. The study was approved by the Ethics Committee of the Central Public Health Laboratory of the Ministry of Public Health and Social Welfare (International Certification FWA No. FWA00020088), under approval code CEI-LCSP 91/010217.

## Results

### Study population characteristics

A total of 737 participants were recruited for the study. The population was predominantly male (85.8%), with a mean age of 32.3 years (SD: 11.0; range: 18–74 years). Most participants were persons deprived of liberty (87.1%). The most frequent risk group was Group 2, representing 71.2% of the sample. Regarding risk factors, tobacco use was the most common (66.9%), followed by drug use (52.3%). Detailed characteristics of the study population are summarized in Table 1.

**Table 1:** Study population characteristics (*N* = 737).

**Characteristic**	**Total (*n* = 737)**
Age (years), mean (SD)	32.3 (11.0)
**Sex**	
Male	632 (85.8)
Female	105 (14.2)
**Participant type**	
Persons Deprived of Liberty (PDL)	642 (87.1%)
Prison staff	95 (12.9%)
**TB risk groups**	
Group 1: Low risk	71 (9.7%)
Group 2: High risk	525 (71.2%)
Group 3: Confirmed TB	67 (9.1%)
Group 4: Previous TB	74 (10.0%)
**Comorbidities and risk factors**	
HIV infection	64 (8.8)
Diabetes	15 (2.1)
Tobacco use	485 (66.9)
Drug use	375 (52.3)

SD, standard deviation.

The prevalence of TB infection was 56.9% (419/737) according to the ELISA test and 57.7% (425/737) according to the FIA test. Overall agreement observed was 92.4% (CI95%: 90.5%−94.5%) between the two assays yielded a Cohen's Kappa value of 0.851 (95% CI: 0.812–0.889; *p* < 0.0001), indicating excellent or almost perfect concordance. A total of 9 participants (1.2%) had at least one indeterminate result and were therefore excluded from concordance analyses. Among them, 6 participants were indeterminate in both assays, 2 were indeterminate only by ELISA, and 1 only by FIA. This distribution explains the 8 indeterminate ELISA results and 7 indeterminate FIA results reported in Table 2.

**Table 2:** Overall agreement between TB-Feron ELISA and TB-Feron FIA (*n* = 728).

**TB-Feron FIA**	**TB-Feron ELISA**			**Total**
	**Positive**	**Negative**	**Indeterminate**	
Positive	395	29	1	425
Negative	25	280	1	305
Indeterminate	0	1	6	7
Total	419	310	8	737
Agreement statistics	Value	CI 95%		
Observed agreement (%)	92.4%	90.5%−94.3%		
Kappa index (κ)	0.851	0.812–0.889		

### Agreement analysis by risk groups

Concordance varied across the four predefined TB risk groups. Moderate agreement was observed in the group without known Group 1, whereas concordance ranged from very good to excellent in the exposed, active TB, and previous TB groups (Table 3).

**Table 3:** Summary of agreement by risk group, sex, and comorbidities.

**Categories**	***N***	**Agreement (%)**	**Positives FIA *n* (%)**	**Positives ELISA *n* (%)**	**Kappa index (κ)**	**CI 95%**
**TB risk groups**						
Group 1: Low risk	71	84.5%	16 (22.9%)	16 (22.9%)	0.570	0.336–0.803
Group 2: High risk	525	93.0%	303 (57.7%)	302 (58.4%)	0.860	0.816–0.903
Group 3: Confirmed TB	67	94.0%	53 (79.1%)	53 (79.1%)	0.809	0.628– 0.991
Group 4: Previous TB	74	94.6%	53 (71.6%)	53 (71.6%)	0.859	0.724–0.993
**Sex**						
Male	632	92.9%	403 (63.8%)	398 (63.0%)	0.845	0.768–0.925
Female	105	91.3%	22 (21.0%)	21 (20.0%)	0.714	0.544–0.928
**Diabetes**						
Yes	64	91.9%	33 (51.6%)	32 (50.0%)	0.820	0.589–1.000
No	665	92.9%	388 (58.3%)	382 (57.4%)	0.851	0.777–0.929
**IV infected**						
Positive	15	80.0%	8 (53.3%)	9 (60.0%)	0.595	0.093–1.000
Negative	709	93.0%	410 (57.8%)	404 (57.0%)	0.852	0.782–0.931
**Tobacco use**						
Yes	485	92.1%	310 (63.9%)	311 (64.1%)	0.823	0.738–0.916
No	240	93.7%	109 (45.4%)	102 (42.5%)	0.867	0.745–0.999
**Drug use**						
Yes	375	92.9%	245 (65.3%)	250 (66.7%)	0.835	0.741–0.944
No	342	92.8%	170 (49.7%)	160 (46.8%)	0.857	0.752–0.964

Although identical overall positivity rates were observed in Group 1 (22.9% for both assays), this masked individual-level discordance, as the 16 FIA-positive and 16 ELISA-positive results did not fully correspond to the same participants. This explains the lower Cohen's kappa value (0.570) despite similar group-level positivity. Nevertheless, overall agreement between the assays in Group 1 was high (84.5%), with discordant results observed in only 11 of 71 samples. Group 1 included four participants living with HIV, among whom only one discordant result was identified, with the FIA yielding a negative result while the ELISA was positive.

Stratified analysis by individual risk factors revealed differences in agreement, particularly among HIV-positive participants. Of the 15 individuals with HIV, 4 belonged to Group 1, 7 to Group 2, and 4 to Group 4. Concordance was notably lower in the HIV-positive group (Kappa: 0.595, moderate) compared to the HIV-negative group (Kappa: 0.852, excellent). Given the small sample size of HIV-positive participants (*n* = 64), we did not perform a sensitivity analysis excluding this subgroup, as the overall concordance analysis already demonstrates robust agreement in the HIV-negative population (*n* = 673, κ = 0.852). The lower concordance observed in HIV-positive individuals may reflect technical variability in measuring low IFN-γ responses rather than true biological differences, as both assays use identical antigens, stimulation protocols, and plasma samples.

No significant differences in concordance levels were found across subgroups defined by diabetes, tobacco use, drug use, or sex.

**Figure 1:**
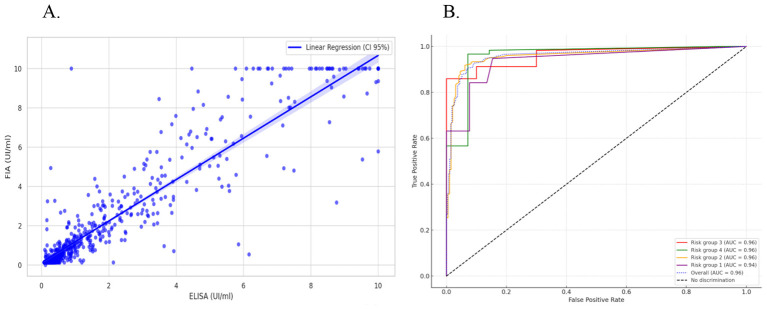
**(A)** Scatter plot of quantitative values for TB antigen in ELISA and FIA tests. The maximum and minimum values represented for both assays are 10 and 0.145 IU/mL, respectively. Pearson's *R* = 0.94 (95% CI: 0.92, 0.96). **(B)** ROC curve of FIA quantitative values against ELISA reference. The graph displays ROC curves in different colors for each risk group, with the blue dotted curve representing the overall test performance.

The Figure 1A shows a strong positive linear correlation (Pearson *R* = 0.94) between the quantitative responses to the TB antigen obtained from the ELISA and FIA tests. This suggests that both quantitative assays yield highly consistent results across the entire dataset, regardless of risk group.

The Figure 1B presents ROC curve analysis evaluating discriminative capacity of the FIA quantitative values using ELISA as a reference. While ELISA is not an independent gold standard, these findings demonstrate that FIA quantitative values consistently predict ELISA-defined positivity across different risk groups, supporting the reliability of FIA classification. The high AUC values indicate that the FIA test performs robustly and consistently across different risk groups.

## Discussion

This is the first study in Paraguay to evaluate the agreement between a fluorescence-based IGRA (STANDARD F TB-Feron FIA) and an ELISA-based IGRA (STANDARD E TB-Feron ELISA) for TB infection detection. The main finding of our study is the excellent overall agreement (κ = 0.851) between these two assays. This high level of concordance aligns with recent studies in other epidemiological settings. For instance, a study in Moldova, a high TB burden country in Eastern Europe, reported near-perfect agreement (κ = 0.91) between these two tests (11). Similarly, research conducted in Indonesia comparing both assays to QuantiFERON-TB Gold Plus found substantial agreement (κ = 0.79) between the FIA and ELISA platforms (12). Our study reinforces this body of evidence and provides the first validation of concordance between these assays in South America prison population—a group disproportionately affected by tuberculosis (15).

It is important to acknowledge that the high concordance observed in our study likely reflects the fact that both assays are manufactured by the same company and use identical antigens (ESAT-6, CFP-10, and TB7.7), blood collection tubes, and incubation protocols. The primary difference lies in the detection method: ELISA uses colorimetric detection with batch processing, while FIA uses fluorescence detection with single-sample processing. The Indonesian study (12), which compared both SD Biosensor assays with QuantiFERON-TB Gold Plus (a different manufacturer using only ESAT-6 and CFP-10 antigens), reported lower concordance (κ = 0.79), suggesting that agreement may be reduced when comparing assays from different manufacturers or with different antigen formulations. A comparison with an independent IGRA platform, such as QuantiFERON or VIDAS TB-IGRA, would have provided a more informative and externally valid assessment of FIA performance (20, 21). Future studies should include head-to-head comparisons with assays from different manufacturers to better understand the interchangeability of IGRA platforms.

This level of concordance is also in line with international literature comparing different IGRA platforms. Studies from South Korea and South Africa evaluating new tests against established assays reported Kappa indices ranging from 0.82 to 0.89 (22). Our findings confirm that FIA technology, despite methodological differences in detection, produces results comparable to ELISA platforms already endorsed by WHO (8). The key advantage of FIA lies in its operational potential: rapid turnaround time and the ability to use portable equipment make it ideal for public health programs in low-resource settings, facilitating decentralized TB infection diagnosis at lower cost (23).

When stratified by the four predefined risk groups, agreement remained strong (Kappa > 0.80) in Groups 2, 3, and 4, suggesting that both tests are particularly reliable for detecting robust immune responses in individuals with documented TB exposure or disease history (24). The lowest concordance (κ = 0.570, moderate) was found in Group 1, composed of individuals with no documented TB case exposure. This is an expected finding, in low-prevalence groups, the contribution of chance agreement is greater, which tends to reduce the Kappa value (25, 26), despite a good agreement. The identical positivity rates in Group 1 (22.9% for both assays) mask individual-level discordance, as the positive results did not always correspond to the same participants. From a programmatic perspective, the FIA appears most useful in Group 2, those exposed to TB and at higher risk of developing active disease. were rapid, decentralized testing could facilitate timely preventive therapy initiation.

A secondary but clinically important finding was the lower concordance observed among HIV-positive participants (κ = 0.595) compared to HIV-negative individuals (κ = 0.852). However, this interpretation must be approached with caution. Given that both assays use identical antigens, stimulation methods, and plasma samples, the observed discordance is far more likely attributable to technical variability in measuring low IFN-γ levels rather than true biological differences between the tests. The small sample size of HIV-positive participants (*n* = 15) limits the statistical power of this subgroup analysis. A major limitation of our study is the absence of CD4+ T-cell count data, which precluded stratified analysis of test performance across different levels of immunosuppression, a factor known to influence IGRA reliability (27). Cellular immunosuppression, particularly low CD4+ counts, may lead to false-negative or indeterminate results in both assays (13, 26, 28, 29). The finding highlights the need for cautious interpretation of IGRA results in immunocompromised populations and supports the development of specific diagnostic algorithms for vulnerable groups, potentially incorporating complementary tests (30).

The STANDARD F TB-Feron FIA offers operational advantages over ELISA that may support its use in decentralized settings. While both assays share the same pre-analytical requirements, ELISA testing requires adequately trained personnel and appropriate instruments and software to perform manual steps, washing procedures, and result interpretation. In contrast, the FIA platform automates the analytical process, directly generating results while reducing hands-on time, allowing single-sample processing, and requiring less laboratory infrastructure through the use of portable equipment. However, the FIA should not be considered a true point-of-care test, as incubation, centrifugation, and trained personnel are still required; its main value lies in facilitating decentralized testing outside centralized reference laboratories (9, 10).

Regarding cost, while the FIA reader is portable and may reduce infrastructure requirements compared to ELISA, we did not conduct a formal cost-effectiveness analysis in this study. The per-test cost of FIA cartridges vs. ELISA kits, as well as the initial investment in equipment, would need to be evaluated in specific programmatic contexts. Established platforms such as QuantiFERON already allow single-sample processing using chemiluminescence technology, offering similar workflow advantages (20, 22). The primary added value of the FIA platform lies in its combination of single-sample capability, reduced hands-on time, and use of portable equipment—features that may facilitate decentralized TB infection screening in resource-limited settings, particularly for contact tracing and prison-based programs.

The main limitation of this study is the absence of a definitive gold standard for tuberculosis (TB) infection diagnosis, as widely acknowledged in the literature and by the World Health Organization (31). TB infection represents a latent state that cannot be directly observed or cultured, precluding the establishment of absolute diagnostic accuracy. Within this framework, we used the WHO-recommended STANDARD E TB-Feron ELISA as an operational reference comparator. Although this assay is robust and well validated, it does not constitute a true gold standard (32). Therefore, comparison with the FIA test provides a practical and meaningful approach for performance evaluation under field conditions.

Additional limitations include the small size of certain subgroups, such as HIV-positive participants, which limited the statistical power of subgroup analyses. Furthermore, quantitative IFN-γ values were capped at 10 IU/mL to limit the influence of extreme values; although analyses without capping were not performed, values above this threshold represented a small proportion of observations, making it unlikely that this approach substantially affected the overall comparative analyses.

Despite these limitations, the comparison of the FIA with a WHO-endorsed ELISA offers pragmatic and relevant validation for real-world use. The strength of our findings is further supported by a large sample size of more than 700 participants, providing robust and reliable evidence.

These results demonstrate that the STANDARD F TB-Feron FIA test is a reliable and comparable alternative to ELISA for detecting TB infection. As authors, we believe that the new diagnostic technique recently available in South America brings benefits for patients with TB infection. First, the lower cost compared to other IGRAs. In addition, in line with another study carried out in Chile, we agree that the technique is simple to implement in medical laboratories (20). Although both assays share the same pre-analytical requirements, including incubation and centrifugation, the analytical phase is markedly faster and operationally simpler for the FIA than for the ELISA (11, 31). Implementing the FIA test could therefore support the expansion of TB screening programs, enabling faster and more efficient identification of individuals (particularly those exposed to TB) for timely initiation of chemoprophylaxis or preventive therapy, contributing to global efforts to end TB.

Our study demonstrates the advantages of the FIA technology in Latin America over other diagnostic platforms, given its operational simplicity, short turnaround time, portability, and reduced infrastructure requirements. These features make it a feasible and scalable solution for implementation, paving the way for broader latent tuberculosis diagnosis and its expansion to diverse healthcare settings across the region. Furthermore, the growing diversity of automated interferon-gamma detection platforms available for TB infection diagnosis will be increasing. Comparative analyzes among FIA-, ELISA-, and chemiluminescence-based assays will be essential to guide optimal test selection across diverse resource settings in the region (20).

## Data Availability

The raw data supporting the conclusions of this article will be made available by the authors, without undue reservation.
